# Chronic pain and opioid misuse: a review of reviews

**DOI:** 10.1186/s13011-017-0120-7

**Published:** 2017-08-15

**Authors:** Pauline Voon, Mohammad Karamouzian, Thomas Kerr

**Affiliations:** 1 British Columbia Centre for Excellence in HIV/AIDS, University of British Columbia, St. Paul’s Hospital, 608-1081 Burrard Street, Vancouver, BC V6Z 1Y6 Canada; 2 School of Population and Public Health, Faculty of Medicine, University of British Columbia, 2206 East Mall, Vancouver, BC V6Z 1Z3 Canada; 3 HIV/STI Surveillance Research Center, and WHO Collaborating Center for HIV Surveillance, Institute for Futures Studies in Health, Kerman University of Medical Sciences, Kerman, Iran; 4 Department of Medicine, University of British Columbia, St. Paul’s Hospital, 608-1081 Burrard Street, Vancouver, BC V6Z 1Y6 Canada

**Keywords:** Chronic pain, Prescription opioid, Substance use, Addiction, Systematic review (3–10 keywords required)

## Abstract

**Objective:**

The crisis of prescription opioid (PO) related harms has focused attention toward identifying and treating high-risk populations. This review aims to synthesize systematic reviews on the epidemiology and clinical management of comorbid chronic pain and PO or other substance misuse.

**Methods:**

A systematic database search was conducted to identify systematic reviews published between 2000 and 2016. Eligible studies were systematic reviews related to chronic non-cancer pain and PO or other substance misuse. Evidence from the included reviews was synthesized according to epidemiology and clinical management themes.

**Results:**

Of 1908 identified articles, 18 systematic reviews were eligible for final inclusion. Two meta-analyses estimated the prevalence of chronic non-cancer pain in individuals using POs non-medically to be approximately 48% to 60%, which is substantially higher than the prevalence of chronic non-cancer pain in general population samples (11% to 19%). Five systematic reviews estimated the rates of PO or other opioid use in chronic pain populations with substantial variation in results (0.05% to 81%), likely due to widely varying definitions of dependence, substance use disorder, misuse, addiction, and abuse. Several clinical assessment and treatment approaches were identified, including: standardized assessment instruments; urine drug testing; medication counts; prescription drug monitoring programs; blood level monitoring; treatment agreements; opioid selection; dosing and dispensing strategies; and opioid agonist treatment. However, the reviews commonly noted serious limitations, inconsistencies, and imprecision of studies, and a lack of evidence on effectiveness or clinical utility for the majority of these strategies.

**Conclusion:**

Overall, current systematic reviews have found a lack of high-quality evidence or consistent findings on the prevalence, risk factors, and optimal clinical assessment and treatment approaches related to concurrent chronic pain and substance misuse. Given the role of systematic reviews in guiding evidence-based medicine and health policy, there is an urgent need for high-quality primary research to guide future systematic reviews to address the escalating epidemic of harms related to chronic pain and substance misuse.

**Electronic supplementary material:**

The online version of this article (doi:10.1186/s13011-017-0120-7) contains supplementary material, which is available to authorized users.

## Background

Across North America, the devastating crisis of prescription opioid (PO) related addiction and overdose has led to escalating mortality rates that have surpassed national mortality rates due to motor vehicle accidents and HIV-related mortality [1, 2]. As such, increased attention is being focused toward understanding the scale of the current epidemic and implementing risk mitigation strategies. In particular, individuals demonstrating concurrent chronic pain and opioid misuse are considered to be at high risk for opioid-related morbidity and mortality [3] given the high risk of opioid dependence among individuals on POs for chronic pain [4] and, conversely, the potential for increased pain severity and decreased pain thresholds among chronic opioid users [5].

The current body of literature on concurrent chronic pain and opioid misuse presents a range of conflicting research on the prevalence, risk factors, and clinical management approaches specific to this growing sub-population. A number of systematic reviews have attempted to synthesize various aspects of the epidemiology or clinical management of comorbid chronic pain and opioid misuse. Given that systematic reviews are the foundation for evidence-based clinical and policy decision-making [6], this ‘review of reviews’ seeks to summarize the findings of published systematic reviews related to the epidemiology, assessment, and treatment of comorbid chronic pain and opioid misuse.

## Methods

### Search strategy

Following the PRISMA guideline [7], we searched for systematic reviews related to chronic non-cancer pain (CNCP) and PO or other opioid misuse that were published in the following databases from January 1, 2000 to October 1, 2016: Medline, Cochrane Library, PsycINFO, Web of Science, EMBASE, and Google Scholar. Search terms were combined using appropriate Boolean operators and included subject heading terms or key words for three key aspects: chronic pain (e.g., pain OR pain management) AND analgesics (e.g., opioid OR opiate OR painkiller OR analgesic) AND abuse (e.g., misuse OR non-medical OR aberrant OR addiction).

### Inclusion and exclusion criteria

Two reviewers independently screened the search results to identify eligible systematic reviews. Reviews were eligible for inclusion if they were: peer-reviewed; systematic reviews related to CNCP and PO or other illicit opioid misuse; focused on adult populations; and published in English. Studies were excluded if they were: non-systematic reviews; reviews of non-primary research (e.g., reviews of clinical guidelines); specific to acute or specialized pain (e.g., cancer pain, terminal or palliate pain); specific to non-adult or specialized settings (e.g., surgical or intensive care units) or populations (e.g., pregnant women, adolescent, elderly, or palliative care populations); or focused on evaluating a specific analgesic brand.

### Data extraction and analysis

Data was extracted and summarized for the following parameters: author name, publication year, journal name, review period, databases used, theme of the review (e.g., epidemiology, assessment, treatment), number of included studies, studied population, and summary of findings. Evidence synthesis was conducted using a narrative approach based on thematic findings.

### Quality assessment

The quality of the systematic reviews was assessed using the Assessment of Multiple Systematic Reviews (AMSTAR) validated scale [8, 9].

## Evidence synthesis

**Fig. 1:**
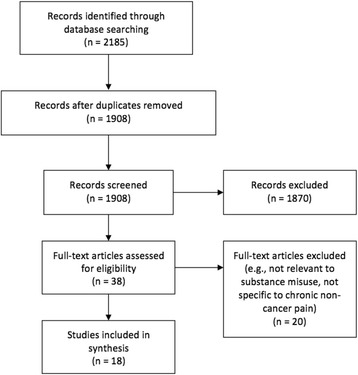
Summary of study identification and selection

From a total of 1908 unique hits, 1870 were removed at the title and abstract screening phase and 38 full-text papers were screened (Fig. 1). Eighteen systematic review articles were eligible for final inclusion and summarized below according to epidemiologic and clinical management themes. Of the eighteen reviews, seven were deemed to be of low quality, six of moderate quality, and five of high quality using AMSTAR scoring (see Additional file 1). Notably, while the search was not restricted to North American studies, only four out of the eighteen reviews were conducted outside of North America, in Europe [10–13].

## Epidemiology of chronic pain and PO use

### Rates of pain in non-medical PO users

#### General population samples

Fischer et al. found a 48% pooled prevalence of pain (95% confidence interval [CI]: 37%–59%; range: 30%–68%) from four general U.S. population samples reporting non-medical PO use (Table 1), although the distinction between chronic versus acute pain was not specified [14]. The authors found significant heterogeneity between studies, likely due to the variability in the populations studied and the measures used to ascertain pain. For instance, the study samples included both younger and older adult populations.

**Table 1:** Rates of pain in samples using prescription opioids (POs) non-medically

Author	Population	Number of studies	Rate calculated	Estimate
Fischer et al. (2012)	General population samples of young adults or adults reporting non-medical PO use	4	Pooled prevalence of pain	48% (95% CI: 37%–59%)
Lusted et al. (2013)	Adult substance use treatment samples reporting non-medical PO use	8	Pooled prevalence of pain	58% (95% CI: 53%–64%)
	Adult substance use treatment samples with PO analgesics reported as the primary drug requiring substance use treatment, or PO dependence reported	7	Pooled prevalence of pain	60% (95% CI: 52%–67%)
	Adult substance use treatment samples with any PO abuse reported	2	Pooled prevalence of pain	50% (95% CI: 40%–60%)

#### Substance use treatment samples

Lusted et al. found a 58% pooled prevalence of pain (95% CI: 53%–64%) from eight samples of adult patients in substance use treatment (Table 1) [15]. Among studies in which POs were reported as the primary drug requiring substance use treatment, or in which PO dependence was reported, the pooled prevalence of pain was slightly higher at 60% (95% CI: 52%–67%). Significant heterogeneity was found between studies, likely due to varying definitions used to ascertain non-medical PO use and pain.

### Rates of dependence, substance use disorder, and problematic use in chronic pain patients

#### Dependence

Minozzi et al. [11] found a 0.5% incidence (range: 0%–24%) and 4.5% prevalence (range: 0%–31%) of opioid dependence syndrome (defined by DSM-IV or ICD-10 criteria) across 17 studies of patients receiving opioids for pain, while Chou et al. [16] found a prevalence of 3%–26% for opioid dependence among chronic pain patients prescribed long-term opioid therapy in primary care settings (Table 2). This substantial heterogeneity in results may be due to several factors. First, low-quality study designs were primarily used, such as uncontrolled case series or cross-sectional designs. Second, there may be significant heterogeneity in the types of populations being studied. For instance, Minozzi et al. found only one study that reported dependence within individuals with a history of substance abuse [17]. Third, the definitions of dependence tend to vary widely across studies. For example, some studies used the DSM-IV definition of opioid dependence, which includes criteria for loss of control over use and negative health or social consequences, while other studies used the ICD-10 definition for opioid dependence, which includes criteria for tolerance, withdrawal, and craving [11]. It is also worth pointing out that ‘opioid dependence,’ which was distinct from ‘opioid abuse’ in the DSM-IV, has been replaced with a single classification of ‘opioid use disorder’ in the revised DSM-5, which may lead to even further variation in future estimates [18].

**Table 2:** Rates of dependence, substance use disorder, and problematic use in samples of chronic non-cancer pain patients

Author	Population	Number of studies	Rate calculated	Estimate
Minozzi et al. (2012)	Individuals receiving any opioid analgesic for acute or chronic pain from any physical condition	17	Median incidence of dependence	0.5% (Range: 0%–24%)
		17	Median prevalence of dependence	4.5% (Range: 0%–31%)
Morasco et al. (2011)	Chronic non-cancer pain patients, regardless of whether they were prescribed opioids	21	Overall prevalence of current substance use disorder	3–48%
		21	Lifetime prevalence of any substance use disorder	16%–74%
Vowles et al. (2015)	Adults with chronic non-cancer pain (≥3 months) using oral opioids	38	Rate of problematic use	<1%–81%
		29	Rate of misuse	21%–29% (95% CI: 13%–38%)
		1	Rate of abuse	8%
		12	Rate of addiction	8%–12% (95% CI: 3%–17%)
Noble et al. (2008)	Patients treated with opioids for chronic non-cancer pain for at least six months	7	Rate of addiction	0.05% (1 out of 2042 patients)
		2	Rate of abuse	0.43% (3 out of 685 patients)
Kalso et al. (2004)	Adult patients with chronic non-cancer pain in randomized controlled trials comparing opioids versus placebo	15	Rate of addiction	Estimates could not be calculated due to small sample sizes and short follow-up periods
Chou et al. (2015)	Adults with chronic pain prescribed long-term opioid therapy – in primary care	3	Prevalence of abuse	0.6%–8%
			Prevalence of dependence	3%–26%
	Adults with chronic pain prescribed long-term opioid therapy – in pain clinics	7	Prevalence of misuse	8%–16%
			Prevalence of addiction	2%–14%

#### Substance use disorder

Morasco et al. was the only review that estimated rates of substance use disorder. Across 21 studies of CNCP patients, the authors found varying prevalence estimates for substance use disorder (not restricted to opioids), ranging from 3% to 48% for current substance use disorders, and 15% to 74% for lifetime history of substance use disorder (Table 2) [19]. The highest rates were observed in individuals seeking opioid prescriptions from emergency departments (74%) [20], individuals with AIDS comorbidity (48%) [21], and individuals who were screened for any substance use using urine toxicology (35%) [22]. However, the authors deemed the quality of the studies to be generally low, and there was substantial heterogeneity in the study settings (e.g., inpatient versus outpatient) and definitions of chronic pain and substance use disorder across studies.

#### Problematic use (e.g., misuse, abuse, and addiction)

The review by Vowles et al. sought to measure more precise estimates of problematic opioid use among adult CNCP patients by using more explicitly defined terms [23]. The three types of problematic use measured were: (1) misuse, defined as the use of opioids contrary to the prescriber’s directions; (2) abuse, defined as opioid use for non-medical intentions (e.g., euphoria or altered consciousness); and (3) addiction, defined as continued opioid use despite impaired control, compulsive use, craving, or demonstrated or potential harms. The overall rate of problematic use across 38 studies ranged from <1% to 81%, with rates of misuse, abuse, and addiction ranging from 21%–29%, 8% (based on one study), and 8%–12%, respectively. In comparison, other reviews found ranges from 8%–16% for opioid misuse [16], 0.43%–8% for opioid abuse [16, 24], and 0.05%–14% for opioid addiction [16, 24] (Table 2). One additional review of randomized controlled trials comparing opioids versus placebo for CNCP was unable to draw conclusions about rates of addiction due to small sample sizes and short follow-up periods across fifteen studies [10]. Factors that may have influenced the variation in these estimates include varying diagnostic criteria for determining problematic use, recruitment methods and exclusion criteria (e.g., many of studies exclude individuals with a history of addiction or substance use from enrollment), lack of comparison groups, high rates of study withdrawal, or lack of intention-to-treat analyses.

### Correlates of concurrent pain and opioid misuse

#### Demographics

Analyzing data from 14 studies, Morasco et al. did not find any demographic factors that were consistently different between CNCP patients with versus without comorbid substance use disorder [19]. Specifically, among CNCP patients, the authors found inconsistent results related to the relationship between substance use status and gender, age, employment, race or ethnicity, marital status, or education status. Conversely, both Chou et al. and Turk et al. found that younger age appeared to increase the risk of opioid misuse [16, 25]. Regarding sex, Turk et al. also found that while female sex did not appear to be a predictor for opioid misuse, there were mixed findings for the effect of male sex [25]. Furthermore, while Turk et al. found mixed results for the effect of race on opioid misuse [25], Cintron et al. found that racial and ethnic minorities (e.g., African Americans, Hispanics) were less likely to misuse POs compared to Caucasian populations, and yet these same racial and ethnic minorities were more likely to experience undertreated pain and less likely to be prescribed opioid analgesics from clinicians [26]. Turk et al. suggest that such mixed findings may be due to inconsistent reporting of demographic variables and underrepresentation of women and racial and ethnic minorities in studies of chronic pain populations [25].

#### Psychiatric comorbidity

Morasco et al. found mixed and inconclusive data on relationship between psychiatric comorbidity and substance use disorder in CNCP patients [19]. Turk et al. found a greater risk of substance abuse in chronic pain patients with mood disorders, particularly unipolar depression, but noted that this was not a consistent predictor across all studies reviewed [25]. Similarly, Chou et al. found that major depression, as well as use of psychotropic medications, were associated with increased risk for opioid misuse among chronic pain patients [16]. In studies of individuals with opioid use disorder, Dennis et al. found that pain was significantly associated with concurrent psychiatric disorder (pooled odds ratio: 2.18; 95% CI: 1.6–2.9; *I* ^2^ = 0.0%) as well as poorer physical, personal, and social functioning compared to individuals with opioid use disorder who did not have pain [27].

#### Substance misuse factors

Several of the reviews found that CNCP patients with a past or present history of opioid or other substance use disorder appear to be at greater risk for PO misuse [12, 16, 19, 25, 28, 29]. Further, the use of multiple substances may be correlated with PO misuse [25, 28]. In individuals with opioid use disorder, Dennis et al. found two studies in which chronic pain had no significant effect on illicit opioid use [27].

#### Treatment-related factors

Morasco et al. found that CNCP patients with comorbid substance use disorder were more likely to be prescribed opioids—and at higher doses—than CNCP patients without substance use disorder [19]. The authors note that this counter-intuitive finding may reflect variation in healthcare setting or comorbid conditions. Based on limited and low-quality data, the authors did not find any significant differences related to treatment response outcomes for CNCP patients with versus without substance use disorder. However, it is important to note that successful treatment of comorbid CNCP and substance use disorder may largely depend on access to opioid maintenance treatment.

## Clinical assessment and management strategies

### Assessment and monitoring

#### Standardized instruments to assess for problematic substance use

A number of standardized instruments have been developed to predict or identify opioid misuse in chronic pain patients (Table 3). Four reviews concluded that there is insufficient evidence to confidently support the accuracy or efficacy of any of these instruments [16, 25, 30, 31]. Specifically, there is a limited number of studies evaluating these instruments, and existing studies tend to be of low to moderate quality with key methodological flaws, such as cross-sectional designs that are unable to determine causality between observed clinical behaviors and subsequent opioid misuse [16, 25, 30, 31]. The validity and reliability of these instruments have been found to be either generally weak or not well evaluated, and potential biases related to patient selection and assessment timing may contribute to inflated estimates of diagnostic accuracy [25, 30]. Furthermore, there is a lack of literature evaluating the comparative utility of these instruments, and the definition of problematic or aberrant behavior varies across each instrument [25, 31].

**Table 3:** Instruments to predict or identify problematic opioid use in individuals with chronic pain

• Current Opioid Misuse Measure (COMM)^ab^
• Opioid Risk Tool (ORT)^a^
• Screener and Opioid Assessment for Patients with Pain (SOAPP) or Screener and Opioid Assessment for Patients with Pain—Revised (SOAPP-R)^a^
• Prescription Drug Use Questionnaire (PDUQ) or Prescription Drug Use Questionnaire—Patient Version (PDUQ-p)^b^
• Pain Medication Questionnaire (PMQ) or Modified Pain Medication Questionnaire (mPMQ)^b^
• Addiction Behaviors Checklist (ABC)^a^
• Prescription Opioid Misuse Index (POMI)^b^
• Pain Assessment and Documentation Tool (PADT)^b^
• Prescribed Opioid Difficulties Scale (PODS)^b^
• Physician Opioid Therapy Questionnaire (POTQ)^a^
• Other instruments to assess substance use not specific to opioids (e.g., DSM-IV, CAGE Questionnaire, Addiction Severity Index, Michigan Alcohol Screening Test, Minnesota Multiphasic Personality Inventory, Screening Instrument for Substance Abuse Potential)

^a^Denotes instruments often used to predict risk of problematic opioid use prior to initiating opioid therapy

^b^Denotes instruments often used to identify current problematic opioid use during ongoing management of opioid therapy

Most importantly, the feasibility, acceptability, and clinical impact of these instruments remain uncertain. First, the instruments may be too lengthy to realistically administer in most clinical settings [30, 31]. Second, the studies of these instruments have limited generalizability, as they have mostly been tested in small, unrepresentative samples within specialized outpatient pain clinic settings with limited representation of women, minority populations, or opioid-naïve patients [25, 31]. Finally, there is a lack of evidence to evaluate the impact these instruments may have on patient outcomes or clinician behaviors [30, 31]. Without strong evidence to guide decision-making based on the results of these instruments, these tools appear to lack significant utility in the clinical setting [30, 31].

#### Urine drug testing

As urine drug testing (UDT) is the most objective method of assessing opioid misuse in chronic pain patients, it is often considered a “gold standard” monitoring approach [25, 29]. However, while UDT may be useful for risk identification and documentation purposes, there remains a lack of strong evidence to support its accuracy or effectiveness in predicting, preventing, or reducing problematic opioid use behaviors or related adverse clinical outcomes (e.g., overdose, mortality) in chronic pain patients [16, 25, 28, 31, 32]. The few studies evaluating UDT for reducing opioid misuse have been found to be poor- to fair-quality observational studies with high risk for bias. For instance, there is substantial heterogeneity across studies in terms of frequency of urine testing, type of urine testing assays performed (e.g., point-of-care immunoassay versus laboratory chromatography), presence of a control group, patient population studied (e.g., certain studies recruited patients who were referred for suspected substance abuse, while other studies excluded individuals with known substance abuse), and other interventions administered alongside urine testing [32]. Additionally, there is high potential for false negative or false positive results that may compromise diagnostic accuracy and the patient-provider relationship [32], and cost-effectiveness and accessibility factors may pose further barriers to clinicians. Given these barriers and the lack of evidence on the clinical impact of UDT on mitigating risk, perhaps it is not surprising that the review by Tournebize et al. estimated that UDT is used for pre-treatment screening and monitoring during opioid treatment by only 15% and 26% of physicians, respectively [13]. Furthermore, even when UDT is used, many prescribers do not accurately interpret the testing results [28, 33].

#### Other assessment and monitoring strategies

There are a variety of other assessment and monitoring strategies intended to mitigate the harms of opioid misuse in chronic pain populations. Additional strategies include medication counts, which involve visual verification to ensure that a patient’s remaining medication supply matches the expected amount remaining on their prescription; prescription drug monitoring programs, which utilize centralized databases to track the opioid prescription history of patients and prescribers; or blood level monitoring to identify opioid misuse, similar to urine drug testing. However, none of the systematic reviews found sufficient reliable data to evaluate the effectiveness of these strategies [16, 28, 31]. Despite this, a growing number of settings are beginning to mandate that clinicians use prescription drug monitoring programs prior to prescribing opioids [34].

### Treatment and intervention approaches

#### Treatment agreements

Treatment agreements—also referred to as treatment contracts or controlled substance agreements—are written documents with stipulations to which the patient formally agrees to prior to initiating opioid treatment. The specific set of terms in a treatment agreement is highly variable, with no strong evidence to support the inclusion or exclusion of particular clauses [32]. Often, the patient must agree to: receive opioid prescriptions from only one physician and pharmacy; not divert their medications; comply with monitoring protocols such as UDT; comply with a prescription refill schedule that does not allow for early refills; and acknowledge that their treatment may be discontinued at the provider’s discretion [28, 32]. While treatment agreements may help promote transparent patient-provider dialogue, and there is some evidence that treatment agreements may promote beneficial clinical outcomes in other patient populations (e.g., addiction, hypertension, obesity) [35], there remains weak and insufficient evidence to support the efficacy of treatment agreements in reducing harmful opioid use behaviors or related adverse clinical outcomes in patients with chronic pain [28, 32]. This lack of evidence to support clinical applicability may be driving low rates of implementation, with an estimated 47% of physicians actually implementing treatment agreements in clinical practice [13].

#### Opioid selection

The review by Argoff et al. highlighted the need for careful attention when prescribing opioids for pain management in individuals with past or present substance misuse [28]. The authors concluded that if opioid treatment is considered in this population (e.g., if non-opioid analgesics have been ineffective for diagnosed pain), weaker opioids (e.g., codeine, tramadol) are preferable to stronger opioids (e.g., oxycodone, hydromorphone), as some evidence has found lower risk for abuse with weaker opioids [28].

Regarding immediate-release formulations, Argoff et al. suggests these may be preferable to extended-release formulations, as there are greater amounts of opioid contained in extended-release formulations that may be associated with higher abuse potential, and there is a paucity of evidence to suggest that long-acting formulations provide superior benefit compared to short-acting formulations [28]. However, Chou et al. noted that the results of research comparing long-acting versus short-acting opioid formulations are inconsistent and difficult to interpret due to variable dosing protocols [16].

Tamper-deterrent formulations are another strategy designed to deter abuse by way of their physical design (e.g., crush-resistant tablets) or ingredients (e.g., nasal irritants to deter snorting, naltrexone to block euphoria from injection, or emulsifying agents to deter injecting). Argoff et al. found some evidence to suggested such formulations can be effective in reducing abuse and tampering, yet other evidence suggests that these formulations are ineffective in that individuals may simply seek other opioids that do not have abuse-deterring properties [28].

#### Opioid dosing and dispensing

One review found weak evidence to suggest that titrating opioids using slow and small dose increases may prevent excessively high doses and promote clinical monitoring of patient responses or potential aberrant behaviors [28]. However, there is a lack of evidence to evaluate the effect of slow and small dose increases on preventing misuse [28]. Given the strong evidence demonstrating the increased risk of overdose and mortality associated with high opioid doses, Argoff et al. found that close monitoring is recommended for patients with high opioid doses, although the definition of what constitutes a ‘high dose’ tends to vary substantially in the literature [28]. Maximum dose restrictions may reduce opioid-related mortality, as suggested by one study of a 120 mg per day morphine-equivalent maximum dose restriction in Washington state that reduced opioid-related mortality by 50% [36], but more research is needed. There is insufficient evidence to evaluate the efficacy of maximum dispensation restrictions on reducing problematic use [31].

#### Opioid agonist treatment

For individuals with CNCP who have or are at risk of opioid addiction, Eilender et al. and other recent studies have found evidence to support the effectiveness of opioid agonist treatment via buprenorphine/naloxone or methadone [29, 37]. Buprenorphine/naloxone may be favourable over methadone due to the lower risk for respiratory depression, potentially lower risk for hyperalgesia or tolerance, and lower dispensing burden, but a downside compared to methadone is that opioids may not be co-prescribed for additional pain control due to the blocking effect of naloxone [29]. A small number of studies have found that individuals may effectively transition from opioid analgesics to buprenorphine/naloxone and achieve reduced pain severity [29, 37], but more research is needed to determine the optimal morphine equivalent dose range prior to buprenorphine/naloxone transition, as higher opioid doses may pose a risk for loss to follow-up due to precipitated withdrawal or other adverse events [29]. Divided doses of buprenorphine/naloxone or methadone may also promote pain control, but there is a limited body of evidence to evaluate this [29].

#### Other treatment approaches

Eilender et al. found a small body of evidence suggesting that psychotherapeutic interventions for concurrent pain and substance misuse using mindfulness or cognitive behavioral therapy approaches may reduce pain severity and opioid misuse [29]. Notably, abstinence-based detoxification treatment is not recommended for CNCP patients with substance use disorders, as it poses high risk for relapse and fatal overdose [38].

## Conclusions

This review has synthesized the findings from 18 systematic reviews related to the epidemiology and clinical management of concurrent CNCP and opioid misuse.

Two meta-analyses estimated the prevalence of CNCP in individuals using POs non-medically to be approximately 48% to 60%, which is substantially higher than the prevalence of chronic pain in general population samples in the United States (11%) and Canada (19%) [39, 40]. Six reviews estimated the rates of dependence, substance use disorder, and problematic opioid use (e.g., misuse, addiction, abuse) in chronic pain patients with highly variable results, which highlights the need for more explicit and consistent definitions of these outcomes in future studies, as well as clear descriptions of the types of populations being studied. In this regard, further research would be useful to determine whether recent changes to the DSM-5 definition of ‘opioid use disorder’, which replaces the previously distinct classifications of ‘opioid dependence’ and ‘opioid abuse’ in the DSM-IV, leads to increased or decreased variation in prevalence estimates.

There are mixed and inconsistent findings regarding demographic factors that may predict opioid misuse in chronic pain populations, perhaps due to inconsistent reporting and underrepresentation of minority demographics [19, 25, 26]. There appears to be a high prevalence of opioid misuse among CNCP patients with psychiatric comorbidity, with potentially greater risk in those with mood disorders, but this evidence is also inconsistent [16, 19, 25, 27]. The most consistent finding across reviews is that a past or present history of opioid or other substance use disorder appears to increase the risk of PO misuse in CNCP patients [12, 16, 19, 25, 28, 29]. More research is also needed on the effect of chronic pain on illicit opioid use in individuals with substance use disorders, the effect of substance use disorders on treatment response outcomes for CNCP patients, and the biological pathways that may exist between these comorbidities.

A number of assessment and monitoring strategies exist to assess for problematic opioid use in CNCP populations, including standardized assessment instruments, urine drug testing, medication counts, prescription drug monitoring programs, and blood level monitoring [25, 28, 30–32]. However, there is a general consensus across reviews that the effectiveness and clinical utility of these strategies for predicting or mitigating problematic opioid use and adverse clinical outcomes have not been well-established, which may in part explain low rates of implementation in clinical settings [13, 16, 25, 28, 30–32]. Similarly, there is a lack of implementation or strong evidence to support the effectiveness of treatment agreements [13, 28, 32].

In terms of other treatment and intervention approaches, there is evidence to support opioid agonist treatment for individuals with chronic pain at risk for problematic drug use, particularly buprenorphine/naloxone [29]. However, more research is needed to determine optimal dosing for patients transitioning from opioid sad for analgesia. Should opioids be prescribed in chronic pain patients at risk for problematic drug use, weaker potency and immediate-release formulations may be preferred [28]. More evidence is needed to determine whether tamper-deterring formulations effectively reduce misuse risk, or if individuals alternate to other drugs that may continue to pose risk. Additionally, greater evidence is needed to evaluate whether slow and small dose increases, maximum dose restrictions, or maximum dispensation restrictions effectively mitigate risky drug behaviors and adverse clinical outcomes.

This review has a number of limitations, including publication bias and varying criteria for inclusion, exclusion and outcome measures across reviews. Additionally, this review excludes findings from primary studies that may have been published after these reviews; however, the intent of this review is to summarize existing syntheses and highlight areas for future systematic review. Nonetheless, to our knowledge, this is the first ‘review of reviews’ to summarize the range of synthesized evidence on various epidemiologic and clinical aspects of concurrent chronic pain and opioid misuse.

In summary, the purpose of this review is to provide a synthesis of systematic reviews related to concurrent chronic pain and opioid misuse, given that this is a sub-population at very high risk for morbidity and mortality, and given that systematic reviews are the basis for evidence-based clinical and policy decision-making. Overall, there is a lack of high-quality evidence or consistent findings on the risk factors for concurrent chronic pain and opioid misuse, as well as clinical approaches to effectively reduce drug-related harms in this population. While updated clinical guidelines have recently been released in an attempt to mitigate the ongoing opioid crisis [3], unfortunately many of the recommendations are based on evidence with serious limitations, inconsistencies, and imprecision as highlighted in this review. Thus, high-quality primary research and comprehensive meta-analyses are urgently needed to guide evidence-based clinical practice to address the escalating public health epidemic of opioid-related harms.

## Additional file

Additional file 1: Methodological quality assessment of the included systematic reviews using AMSTAR scoring. (DOCX 26 kb)

## Abbreviations

AIDS: Acquired immune deficiency syndrome
AMSTAR: Assessment of Multiple Systematic Reviews
CNCP: Chronic non-cancer pain
DSM-IV: Diagnostic and Statistical Manual of Mental Disorders, 4th Edition
ICD-10: International Statistical Classification of Diseases and Related Health Problems, 10th Revision
PO: Prescription opioid
PRISMA: Preferred Reporting Items for Systematic reviews and Meta-Analyses
UDT: Urine drug testing
