# Supplementary material for: Chronic pain and opioid misuse: a review of reviews
Source: Subst Abuse Treat Prev Policy. 2017 Aug 15;12:36. doi: 10.1186/s13011-017-0120-7 (PMC5558770; doi:10.1186/s13011-017-0120-7)
Supplement: Additional file 1: — Methodological quality assessment of the included systematic reviews using AMSTAR scoring. (DOCX 26 kb) [file 13011_2017_120_MOESM1_ESM.docx]

| **Study** |  | **1** | **2** | **3** | **4** | **5** | **6** | **7** | **8** | **9** | **10** | **11** | **Rating Score*** | **Overall Quality^δ^** |
| --- | --- | --- | --- | --- | --- | --- | --- | --- | --- | --- | --- | --- | --- | --- |
| Kalso et al. 2004 | | No | Yes | Yes | No | No | Yes | Yes | Yes | No | No | No | 5 | Low |
| Cintron et al. 2006 | | No | C/A | No | No | No | Yes | No | No | C/A | N/A | No | 2 | Low |
| Noble et al. 2008 | | No | Yes | Yes | Yes | No | Yes | Yes | Yes | Yes | Yes | Yes | 9 | High |
| Turk et al. 2008 | | No | Yes | No | No | No | Yes | No | No | C/A | N/A | No | 2 | Low |
| Chou et al. 2009 | | No | Yes | No | No | Yes | Yes | Yes | No | No | N/A | No | 4 | Low |
| Starrels et al. 2010 | | No | Yes | Yes | No | No | Yes | Yes | Yes | N/A | No | Yes | 6 | Moderate |
| Morasco et al. 2011 | | No | Yes | Yes | No | No | Yes | Yes | Yes | N/A | N/A | Yes | 6 | Moderate |
| Fischer et al. 2012 | | No | Yes | Yes | Yes | No | Yes | Yes | Yes | Yes | Yes | Yes | 9 | High |
| Becker et al. 2013 | | No | Yes | Yes | No | No | Yes | Yes | Yes | No | N/A | No | 5 | Low |
| Lusted et al. 2013 | | No | Yes | Yes | No | No | Yes | No | C/A | Yes | Yes | Yes | 6 | Moderate |
| Minozzi et al. 2013 | | No | Yes | Yes | Yes | Yes | Yes | Yes | Yes | N/A | N/A | Yes | 8 | High |
| Argoff et al. 2014 | | No | C/A | No | Yes | No | Yes | Yes | Yes | Yes | N/A | No | 5 | Low |
| Chou et al. 2015 | | Yes | Yes | Yes | No | Yes | No | Yes | Yes | Yes | Yes | Yes | 9 | High |
| Dennis et al. 2015 | | Yes | Yes | Yes | Yes | No | Yes | Yes | Yes | Yes | Yes | Yes | 10 | High |
| Vowles et al. 2015 | | No | Yes | Yes | No | No | Yes | Yes | Yes | Yes | N/A | Yes | 7 | Moderate |
| Eilender et al. 2016 | | Yes | Yes | No | No | No | Yes | No | No | N/A | N/A | No | 3 | Low |
| Timmerman et al. 2016 | | Yes | Yes | Yes | No | No | Yes | Yes | Yes | N/A | N/A | Yes | 7 | Moderate |
| Tournebize et al. 2016 | | No | Yes | Yes | Yes | No | Yes | Yes | Yes | N/A | N/A | No | 6 | Moderate |
| Total % of reviews meeting each criterion | | 22% | 88% | 72% | 33% | 16% | 94% | 77% | 72% | 38% | 27% | 55% | Median Score = 6 |  |

* AMSTAR contains the following 11-items to appraise the methodological aspects of the systematic reviews: 1. ‘a priori’ design provided; 2. duplicate study selection/data extraction; 3. comprehensive literature search; 4. status of publication as inclusion criteria (i.e., grey or unpublished literature); 5. list of studies included/excluded provided; 6. characteristics of included studies documented; 7. scientific quality assessed and documented; 8. appropriate formulation of conclusions (based on methodological rigor and scientific quality of the studies); 9. appropriate methods of combining studies (homogeneity test, effect model used and sensitivity analysis); 10. assessment of publication bias (graphic and/or statistical test); and 11. conflict of interest statement. All 11-items were scored as “Yes”, “No”, “Can’t Answer (C/A)” or “Not Applicable (N/A)”.

**^δ^** High Quality = >70% points; Moderate Quality = 50-70% points; Low Quality = <50% points.
